# Atypical MRI Presentation of High-Grade Chondrosarcoma in the Proximal Femur

**DOI:** 10.5334/jbsr.3063

**Published:** 2023-02-22

**Authors:** Nico Hustings, Valerie Van Ballaer, Geert Vanderschueren

**Affiliations:** 1 UZ Leuven, BE

**Keywords:** MRI, CT, radiography, pathology, chondrosarcoma, bone lymphoma, bone tumor

## Abstract

**Teaching Point:** Although magnetic resonance imaging (MRI) plays a considerable role in the detection and differentiation of chondrosarcoma (e.g., by cortical breakthrough, peritumoral soft tissue edema, and extra-osseous extension), it is important to be aware of atypical features of common bone tumors.

## Case History

**Figure 1:**
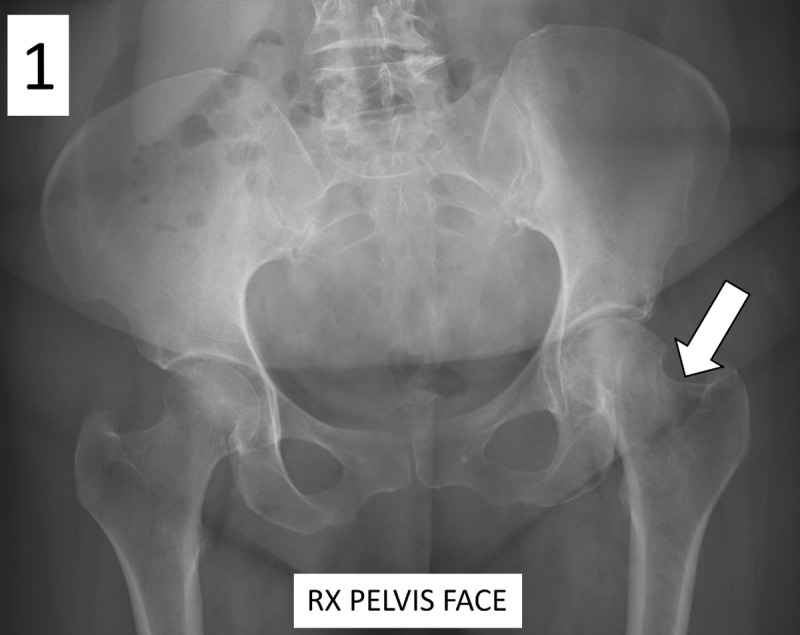


A 60-year-old woman presented with chronic pain in the left hip. For assumed osteo-arthritis, X-rays were ordered. The pelvic radiograph showed a blurry outlined osteolucent lesion in the left femoral neck, with focal broadening of bone (Figure 1, arrow).

**Figure 2:**
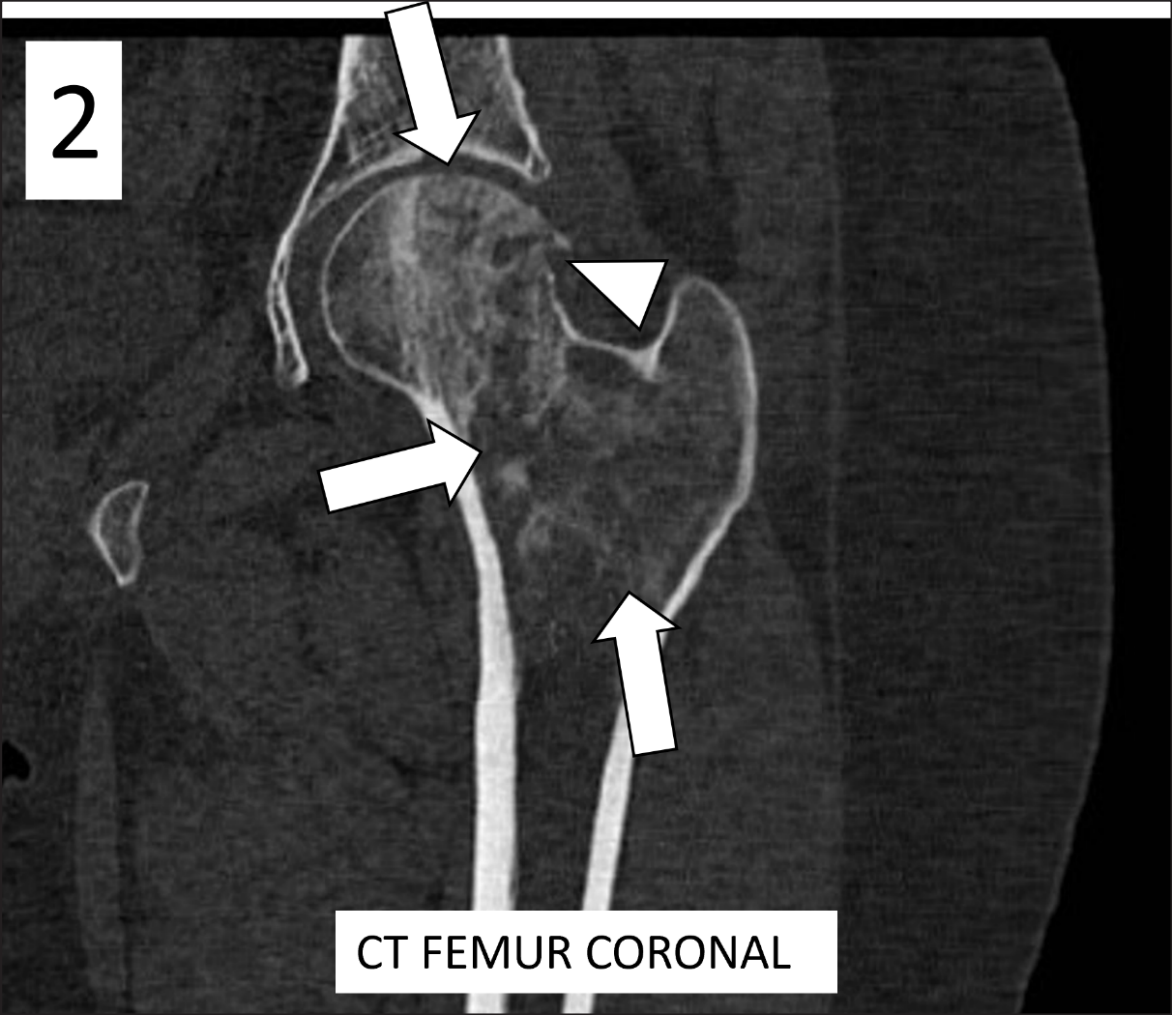


Computed tomography (CT) revealed moth-eaten osteolysis in the femoral head with extension to the intertrochanteric region (Figure 2, arrows), accompanied by endosteal scalloping of the cortex and focal cortical breakthrough (Figure 2, arrowhead).

**Figure 3:**
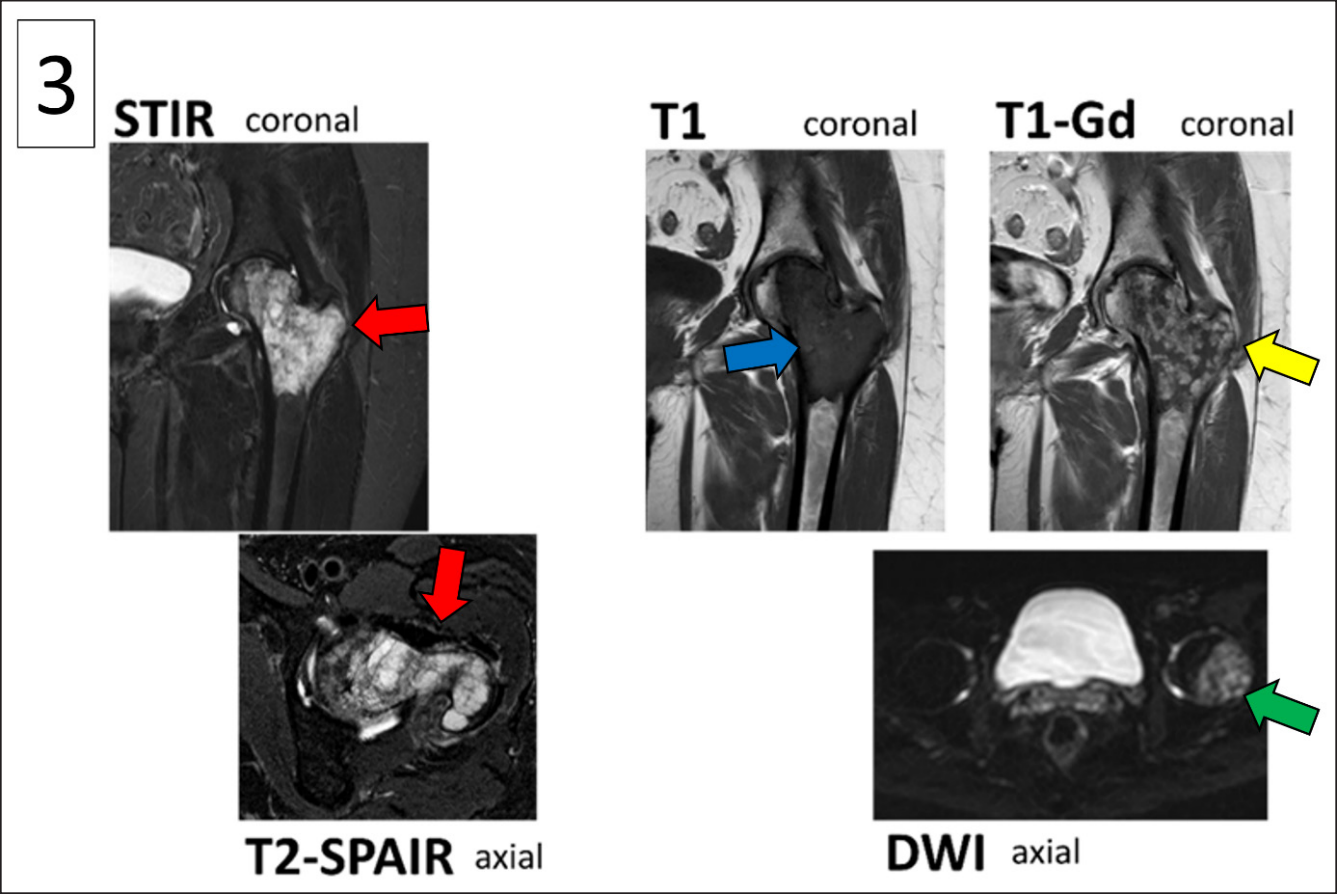


Magnetic resonance imaging (MRI) confirmed a lesion in the proximal femur with remarkably limited extra-osseous extension and limited edema in the surrounding soft tissues (Figure 3). On STIR and T2-SPAIR images the lesion showed high signal, with inlying foci of low signal (Figure 3, red arrows). On T1-WI the lesion demonstrated low signal (Figure 3, blue arrow) with nodular contrast enhancement pattern seen on post-contrast T1-WI images (Figure 3, yellow arrow). The lesion also exhibited diffusion restriction (Figure 3, green arrow).

Regarding differential diagnosis, our first consideration was primary bone lymphoma, because of the typical location in the proximal femur and the presence of cortical breakthrough. Low-grade chondrosarcoma was also included in the differential, due to the hyperintense appearance on T2-WI and the nodular contrast enhancement. We considered a high-grade chondrosarcoma or osteomyelitis to be less likely, because of the limited soft tissue extension and edema. However, on histopathology the final diagnosis turned out to be a high-grade chondrosarcoma.

## Comments

Chondrosarcoma is the second most common malignant bone tumor after osteosarcoma, with an incidence of 1/200,000 per year [1]. The tumor is classified into three grades according to the degree of malignancy: low, intermediate, and high. This differentiation in grades is important because of the difference in prognosis and therapy. Low-grade chondrosarcoma is usually treated by surgical removal followed by adjuvant radiotherapy.

The other two grades of chondrosarcoma are more likely to metastasize, with reported incidences of 30% (intermediate-form) up to 70% (high-grade form). The therapeutic approaches for these tumors are amputation or endoprosthetic reconstruction combined with limb salvage techniques. MRI plays an important role in the detection and differentiation of these tumors because of the accurate detection of cortical destruction, peritumoral soft tissue edema, and soft tissue tumor extension. However, it has been reported that the combined use of MRI and radiography results in higher accuracy, because radiographs are more sensitive in the detection of calcification, scalloping, and cortical penetration [1]. Although literature reports characteristics that can differentiate between the different grades of chondrosarcomas, atypical presentation may still occur – like in our case – and definite diagnosis is made on histopathology [1].
